# Validation of diffusion tensor MRI measurements of cardiac microstructure with structure tensor synchrotron radiation imaging

**DOI:** 10.1186/s12968-017-0342-x

**Published:** 2017-03-10

**Authors:** Irvin Teh, Darryl McClymont, Marie-Christine Zdora, Hannah J. Whittington, Valentina Davidoiu, Jack Lee, Craig A. Lygate, Christoph Rau, Irene Zanette, Jürgen E. Schneider

**Affiliations:** 1 Division of Cardiovascular Medicine, Radcliffe Department of Medicine, University of Oxford, Oxford, UK; 2 Leeds Institute of Cardiovascular & Metabolic Medicine, University of Leeds, Leeds, UK; 3 Diamond Light Source, Didcot, UK; 4 Department of Physics and Astronomy, University College London, London, UK; 5 Division of Imaging Sciences and Biomedical Engineering, King’s College London, London, UK; 6 University of Manchester, Manchester, UK; 7 Feinberg School of Medicine, Northwestern University, Chicago, USA

**Keywords:** Diffusion tensor imaging, Structure tensor, Synchrotron radiation imaging, Cardiovascular magnetic resonance, Validation, Tissue microstructure

## Abstract

**Background:**

Diffusion tensor imaging (DTI) is widely used to assess tissue microstructure non-invasively. Cardiac DTI enables inference of cell and sheetlet orientations, which are altered under pathological conditions. However, DTI is affected by many factors, therefore robust validation is critical. Existing histological validation is intrinsically flawed, since it requires further tissue processing leading to sample distortion, is routinely limited in field-of-view and requires reconstruction of three-dimensional volumes from two-dimensional images. In contrast, synchrotron radiation imaging (SRI) data enables imaging of the heart in 3D without further preparation following DTI. The objective of the study was to validate DTI measurements based on structure tensor analysis of SRI data.

**Methods:**

One isolated, fixed rat heart was imaged ex vivo with DTI and X-ray phase contrast SRI, and reconstructed at 100 μm and 3.6 μm isotropic resolution respectively. Structure tensors were determined from the SRI data and registered to the DTI data.

**Results:**

Excellent agreement in helix angles (HA) and transverse angles (TA) was observed between the DTI and structure tensor synchrotron radiation imaging (STSRI) data, where HA_DTI-STSRI_ = −1.4° ± 23.2° and TA_DTI-STSRI_ = −1.4° ± 35.0° (mean ± 1.96 standard deviation across all voxels in the left ventricle). STSRI confirmed that the primary eigenvector of the diffusion tensor corresponds with the cardiomyocyte long-axis across the whole myocardium.

**Conclusions:**

We have used STSRI as a novel and high-resolution gold standard for the validation of DTI, allowing like-with-like comparison of three-dimensional tissue structures in the same intact heart free of distortion. This represents a critical step forward in independently verifying the structural basis and informing the interpretation of cardiac DTI data, thereby supporting the further development and adoption of DTI in structure-based electro-mechanical modelling and routine clinical applications.

**Electronic supplementary material:**

The online version of this article (doi:10.1186/s12968-017-0342-x) contains supplementary material, which is available to authorized users.

## Background

Diffusion magnetic resonance imaging (MRI) [1] is a valuable method for characterising the three-dimensional (3D) microstructure in biological tissues. It is finding increasing application in the heart, and has been used to assess cardiac hypertrophy [2–4], myocardial infarction [5, 6], myocardial fibrosis [7], cardiac contraction [8–10] and inform on patient-specific tissue mechanical properties [11]. In particular, diffusion tensor imaging (DTI) [12] enables estimation of principal eigenvectors of the diffusion tensor (DT), **v**_**1**_, **v**_**2**_ and **v**_**3**_, that are understood to correspond to the cardiomyocyte (hereinafter referred to as ‘cell’) long-axis, sheetlet and sheetlet-normal directions respectively [13, 14]. However, diffusion in biological tissue is affected by tissue heterogeneity and complexity, leading to ambiguity in the diffusion MRI signal.

A primary objective of cardiac DTI is the assessment of the 3D arrangement of cardiomyocytes in the intact organ. Recent in vivo cardiac DTI in rats [15] and humans [16] employed resolutions of 0.35 × 0.35 × 3 mm and 2.8 × 2.8 × 8 mm respectively. This translates to averaging over large numbers of cardiomyocytes within each voxel, on the order of 1 × 10^4^ and 1 × 10^6^ respectively, based on histological measurements [17–19]. In fitting a single tensor to the DTI data, the structural complexity of the myocardium within each voxel, comprising multiple cell types and orientations, is considerably reduced into three orthogonal eigenvectors and eigenvalues, that assumes unrestricted diffusion in a single compartment.

Diffusion MRI is a sensitive but non-specific marker of tissue microstructure. Besides intra-voxel averaging, water diffusion in the heart is affected by cell size, shape, permeability, and the presence of structures including vessels, collagen fibres and extracellular fluid. In vivo DTI is additionally subject to major challenges of cardiac and respiratory motion, strain, perfusion and heart rate variation. Technical considerations include the signal-to-noise ratio, temperature, eddy currents and the choice of pulse sequence, b-value, diffusion gradient duration, diffusion time and data fitting routine. Furthermore, limited imaging resolution leads to partial volume, and DTI is a simplified representation that assumes that diffusion is unrestricted and follows a Gaussian distribution. For these reasons, it is important that DTI measurements are independently validated.

One study in bovine heart made use of ink prints and optical scanning at 42 μm in-plane resolution to validate DTI measurements [20]. Histological methods additionally offer sub-micrometre in-plane resolutions and cell-specific staining for more accurate assessment of microstructure [14, 21, 22]. Polarised light imaging exploits the birefringence properties of myosin for estimation of cell orientation, but is limited by imaging resolution and range of resolvable orientations [23]. These two-dimensional (2D) methods typically require harsh preparation involving ethanol dehydration, wax or polymer embedding and mechanical slicing. Cell shrinkage, protein denaturation due to hot wax or exothermic polymer curing, and damage to individual slices due to imperfect embedding or slicing are common issues. Assessment of cell orientations in three dimensions and reconstruction of 3D volumes from 2D imaging slices in the presence of variable distortions remains challenging. These methods are also limited in FOV, labour-intensive and destructive in nature. Scanning electron microscopy [24], confocal microscopy [25] and light sheet fluorescent microscopy [26] enable high resolution imaging in three dimensions over fields-of-view that can be stitched together. However, the total FOV remains small and they require similar preparation steps as other 2D methods. A major confound with the approaches described is that the sample is in a different physical state between DTI and the technique used for validation. This imposes marked sensitivity to the reconstruction and registration methods used, and compromises the accuracy of validation. Anatomical MRI and computed tomography with X-ray laboratory sources circumvent these issues, but face insufficient resolutions in the order of 50 μm [27] and 26 μm [28] respectively, rendering cells indiscernible and thereby limiting their utility as validation techniques.

Conventional radiography is performed with laboratory sources. However, scan times for acquiring high resolution data are prohibitive. Furthermore, data are typically acquired in absorption mode, and small density differences in soft tissues yield poor contrast. A synchrotron is a large-scale facility that provides high-brilliance X-ray beams. These can be conditioned to obtain highly coherent beams of high flux, facilitating implementation of X-ray phase-contrast imaging methods. In the X-ray energy range used for imaging, X-rays are phase shifted much more than they are absorbed. Phase information can therefore provide superior contrast to conventional absorption imaging, allowing discrimination of features invisible in absorption contrast. Detectors, however, can only measure intensities and are unable to detect the phase shift of the incoming X-ray beam directly. Therefore, the phase shift needs to be transformed into intensity modulations in the detector, typically through interference effects. Several imaging techniques utilising phase contrast have been developed. The free space propagation method is employed in this study due to its relative ease in experimental setup and data analysis [29].

Synchrotron radiation imaging (SRI) offers both excellent resolution and coverage, without further sample preparation. Recent studies acquired whole foetal rabbit heart data [30] with a FOV of 10 × 10 × 20 mm and whole mouse heart data [31] with a FOV of 15.2 × 15.2 × 3.7 mm, both with an acquired isotropic spatial resolution of 15 μm. The high brilliance and flux of synchrotron radiation and use of phase contrast reconstruction obviated the need for contrast agents. In this study, we performed DTI, followed by structure tensor synchrotron radiation imaging (STSRI) in a whole ex vivo rat heart at an acquired isotropic spatial resolution of 7.2 μm. We show excellent correspondence of DT and ST eigenvectors with the cardiomyocyte long-axis, and propose STSRI as a gold standard for validation of DTI in the whole organ.

## Methods

### Sample preparation

Two healthy female Sprague–Dawley rats were anaesthetised with sodium pentobarbital (55 mg/kg I.P.) and heparin (300 IU). The hearts were rapidly excised then swiftly perfused and cardioplegically arrested in Langendorff constant pressure mode at 80 mmHg with oxygenated (95% O_2_/5% CO_2_) modified Krebs-Henseleit solution at 37 **°**C (mM): NaCl 118.5, NaHCO_3_ 25.0, KCl 4.75, MgSO_4_.7H_2_O 1.22, KH_2_PO_4_ 1.21, Glucose 11.0, CaCl_2_.2H_2_O 1.84 and high potassium solution (mM): NaCl 125.0, KCl 20.0, MgCl_2_.6H_2_O 1.0, HEPES 5.0, Glucose 11.0, CaCl_2_.2H_2_O 1.8 respectively. The hearts were perfused at 5 ml/min for 20 min via peristaltic pump (Gilson, WI, USA) with low osmolality (300 ± 10 mOsm) Karnovsky’s fixative (%): paraformaldehyde 0.45, glutaraldehyde 0.57, sodium cacodylate 0.97 doped with 2 mM gadolinium complex Prohance (Bracco, MN, USA). The hearts were then immersed in 50 mL of the same fixative and kept at 4 °C for 4 days to continue fixation. The fixative was changed on day 2. Prior to MRI, samples were rinsed three times in PBS + 2 mM gadolinium, and embedded in 1% agarose gel (Web Scientific, Crewe, UK) in PBS + 2 mM gadolinium to minimise sample motion and gradients in osmolality and contrast agent concentration. Following MRI, the samples were kept at 4 °C then scanned with synchrotron radiation imaging (SRI).

### Diffusion tensor imaging

Non-selective 3D fast spin echo DTI data were acquired on a 9.4 T preclinical MRI scanner (Agilent, CA, USA) with shielded gradients (max gradient strength = 1 T/m, rise time = 130 μs) and a quadrature-driven birdcage coil (Rapid Biomedical, Rimpar, Germany) of inner diameter = 20 mm. Imaging parameters were: TR/TE = 250/9.5 ms, echo spacing = 5.1 ms, echo train length = 8, matrix = 216 × 144 × 144, field-of-view = 21.6 × 14.4 × 14.4 mm, isotropic resolution = 100 μm, centric phase encoding, receiver bandwidth = 100 kHz, number of diffusion-weighted (DW) directions = 30, number of non-DW images = 4, diffusion gradient duration = 2 ms, diffusion time = 5.5 ms, b = 1,000 s/mm^2^, and acquisition time = 6 h 7 min.

### Synchrotron radiation imaging

**Fig. 1:**
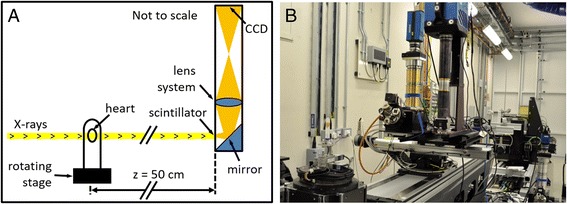
**a** Schematic diagram and **b** photograph of the SRI setup. The coherent X-ray beam passes through the heart and into the detector, which is comprised of a scintillator, that converts X-rays into visible light, a mirror, lens system and charge-coupled device (CCD) camera

SRI data were acquired at beamline I13-2 (imaging branch) of the Diamond Light Source (Didcot, UK; Fig. 1) [32]. In-line phase contrast imaging [33] and tomography were performed using a polychromatic beam from an undulator. The undulator gap was 6 mm and the current in the electron storage ring was 300 mA. To reduce energy deposition in the sample, low-energy X-rays were filtered with 0.3 mm steel and 0.7 mm aluminium leading to an X-ray spectrum with harmonics in the range of approximately 15–30 keV. An average energy of 20 keV was assumed. Three thousand and one projection images with uniform angular spacing were acquired over a long-axis rotation of 180° using a scintillator-coupled PCO 4000 camera with sensor size = 2672 (h) × 4008 (w) pixels and pixel size = 9.0 μm. The following parameters were used to image the first heart: total magnification = 2.5×, acquired isotropic spatial resolution = 7.2 μm, reconstructed pixel size = 3.6 μm, exposure time = 4.0 s and propagation distance, z = 50 cm. Three overlapping short-axis slabs were acquired to span the whole left ventricle (LV) and right ventricle (RV) with combined field-of-view = 17.3 × 14.0 × 14.0 mm and total acquisition time = 10 h. A single region-of-interest in the apical LV myocardium was imaged in the second heart at higher resolution: total magnification = 8×, acquired isotropic spatial resolution = 2.2 μm, reconstructed pixel size = 1.1 μm, number of projections = 4001 and acquisition time = 4 h 20 min. The parameters were chosen to optimise the image quality and to limit radiation damage. Flat-field (background illumination) and dark image correction was performed on the projection data. To enhance soft-tissue contrast, a single-distance phase retrieval algorithm [34] was applied to the projection data (Eq. 1). Subsequently, the 3D phase volume was reconstructed using filtered back-projection [35].

1 $$ \upvarphi \left( x, y\right)=\frac{\delta_{\lambda}}{2{\beta}_{\lambda}} \ln \left({\mathrm{F}}^{-1}\left\{\frac{\mathrm{F}\left[\mathrm{I}\left( x, y\right)/{\mathrm{I}}_0\left( x, y\right)\right]}{1+\left(\lambda z{\delta}_{\lambda}/4\uppi {\beta}_{\lambda}\right)\left({u}^2+{v}^2\right)}\right\}\right) $$

where φ(*x,y*) is the phase shift of the X-rays in the sample, F is the Fourier transform, I_0_(*x,y*) and I(*x,y*) are the intensities before and after passing through the sample, *λ* is the wavelength and was set to 6.2 × 10^−11^ m. *δ*_*λ*_ is the refractive index decrement which indicates how much X-rays are refracted by the sample, and *β*_*λ*_ is the extinction coefficient which indicates how much X-rays are absorbed by the sample. *δ*_*λ*_/*β*_*λ*_ was set to 1000 based on visual optimisation of image sharpness and contrast. u and v are the Fourier coefficients or spatial frequency coordinates, and z is the propagation distance.

### Tensor analysis

Diffusion and structure tensors were calculated from DTI and SRI data respectively. 3D tensors were fitted to DTI data, including all DW and non-DW volumes, using non-linear least squares. The mean ADC was calculated as the mean of the principal eigenvalues. The segmentation of the heart consisted of all voxels with signal intensity in the non-DW images > 20% of the global maximum, and mean ADC < 1.8 × 10^−3^ mm^2^/s. The heart was rigidly registered using cubic interpolation, so that the lateral-septal, anterior-posterior and apico-basal orientations corresponded to the x, y and z directions respectively. The transformation, **M** was calculated based on the long-axis vector, determined by linearly fitting the centres-of-mass of the segmented LV cavity in 2D short-axis planes, and two manually-specified points at the intersections of the LV and RV at the anterior and posterior walls in a mid-ventricular slice.

The SRI images were first manually registered in 2D to the non-DW MRI data by rigid body rotation with linear interpolation of the SRI short-axis slices. STs were generated based on the signal intensity gradients in the SRI phase contrast images using the method of quadrature filters (Eq. 2) [36].

2 $$ \mathbf{T}={\displaystyle {\sum}_k}\parallel {q}_k\parallel \left({\mathbf{n}}_k{\mathbf{n}}_k^T - \frac{\mathbf{I}}{m-1}\right) $$

where **T** is the structure tensor, *q*_*k*_ is the output from quadrature filter, **n**_*k*_ is the orientation of quadrature filter *k*, *m* is the dimensionality of **T**, and **I** is the identity tensor.

Here, SRI images were convolved with six quadrature filters in 3D using freely available Matlab code [37] yielding a symmetric 3 × 3 tensor, **T**, at every voxel. The centre frequency = $\frac{\pi}{3\sqrt{2}}$, bandwidth = 2 octaves and kernel size = 11^3^ voxels. A kernel size of 7^3^ voxels, with matching centre frequency of $\frac{2\pi}{3}$, was also used to examine the effect of varying the kernel size. This was further explored in the higher-resolution data acquired in the second heart by setting bandwidth = 2 octaves, and jointly varying the centre frequency = $\frac{2\pi}{3},\frac{\sqrt{2}\pi}{3},\ \frac{\pi}{3},\ \frac{\pi}{3\sqrt{2}},\ \frac{\pi}{6}$ and kernel size = 7^3^, 7^3^, 9^3^, 11^3^, 17^3^. The STs were spatially averaged element-wise via convolution with a 7^3^ voxel Gaussian averaging filter. STs were then downsampled to the resolution of the DTI data by calculating the mean of each element in 28^3^ voxel neighbourhoods. The downsampled 3D structure tensor (ST) volume was registered to the transformed DTI data by application of the transformation matrix, **M**. Individual STs were likewise rotated.

### Simulations

A structured image volume was generated to investigate the behaviour of the ST reconstruction with respect to the underlying microstructure. The simulation comprised a 0.9 × 0.9 × 0.1 mm transmural block of close-packed elongated cuboidal cells generated at 3.6 μm isotropic resolution. The cuboid thickness was specified to match the DTI voxel size, while the in-plane dimensions were defined to clearly illustrate the transition in HA. The simulated cuboidal cells were organised into groups [24], or sheetlets, 3 cells deep and uniformly aligned within each sheetlet. Helix angle (HA) across sheetlets varied linearly with transmural position, from −88° in the subepicardium to 88° in the subendocardium. Transverse angle (TA) was set to 0°; these are described in detail in *Angle Mapping*. The following parameters were simulated based on the SRI data in the heart as well as literature values [17–19]: cell length = 108 μm, cell thickness = 14 μm, cell width = 18 μm, gap thickness between sheetlets = gap width between cells = 7 μm, gap thickness between cells = 4 μm, intracellular signal intensity (in arbitrary units), S_intracellular_ = 50 and extracellular signal intensity, S_extracellular_ = 100. Poisson noise was subsequently added, where standard deviation of i^th^ voxel = √S_i_. STs were reconstructed at 100 μm isotropic resolution as described in *Tensor Analysis.* For comparison, DTs were generated by setting **v**_**1,DT**_, **v**_**2,DT**_ and **v**_**3,DT**_ to the cell long-axis, sheetlet and sheetlet-normal directions.

### Angle mapping

Local coordinate systems were defined in voxels across the myocardium based on Laplace’s method [38, 39]. Maps of HA, TA, SE and SA were calculated in the DTI and structure tensor synchrotron radiation imaging (STSRI) data with reference to the local coordinate systems. HA is the angle subtended by the projection of **v**_**1,DT**_/**v**_**3,ST**_ onto the tangential-longitudinal plane and the short-axis plane; TA is the angle subtended by the projection of **v**_**1,DT**_/**v**_**3,ST**_ onto the short-axis plane and the local circumferential axis; SE is the angle subtended by the projection of **v**_**3,DT**_/**v**_**2,ST** (3.6μm)_/**v**_**1,ST** (1.1μm)_ onto the radial-longitudinal plane and the short-axis plane; SA is the angle subtended by the projection of **v**_**3,DT**_/**v**_**2,ST** (3.6μm)_/**v**_**1,ST** (1.1μm)_ onto the short-axis plane and the local radial axis. Transmural angular profiles were measured in the whole LV, and results are presented segmented according to the 17-segment American Heart Association heart model [40].

### Tractography

Tracking of DTI and STSRI eigenvectors **v**_**1**_, **v**_**2**_ and **v**_**3**_ was performed in the whole heart using Diffusion Toolkit [41]. Here we refer to ‘tracks’ as streamlines connecting contiguous tensors with similar alignment. Tracks were generated from all voxels within the segmented heart volume, using a 2^nd^ order Runge–Kutta method, spline filter, an angle threshold of 30° and no FA threshold. A minimum length threshold of 0.5 mm was used for **v**_**1,DT**_/**v**_**3,ST**_ tracks. Tracks are displayed in the whole heart and in a mid-ventricular short-axis slice using Trackvis [41], and colour-coded by orientation: apico-basal (red), anterior-posterior (green) and lateral-septal (blue).

### Effect of vasculature

The effect of vessels on the ST reconstruction was investigated in the higher resolution SRI data. Vessels, including their immediate neighbourhood, were segmented by thresholding the SRI images. Voxels with magnitude ≥ 0.006 were retained and dilated with a kernel of radius = 4.4 μm. Connected component analysis was performed [42], and components with a volume smaller than 6.5 × 10^4^ μm^3^ were discarded. Voxel-averaged STs were calculated excluding contributions from voxels corresponding to the segmented vessels.

## Results

### Experimental data

**Fig. 2:**
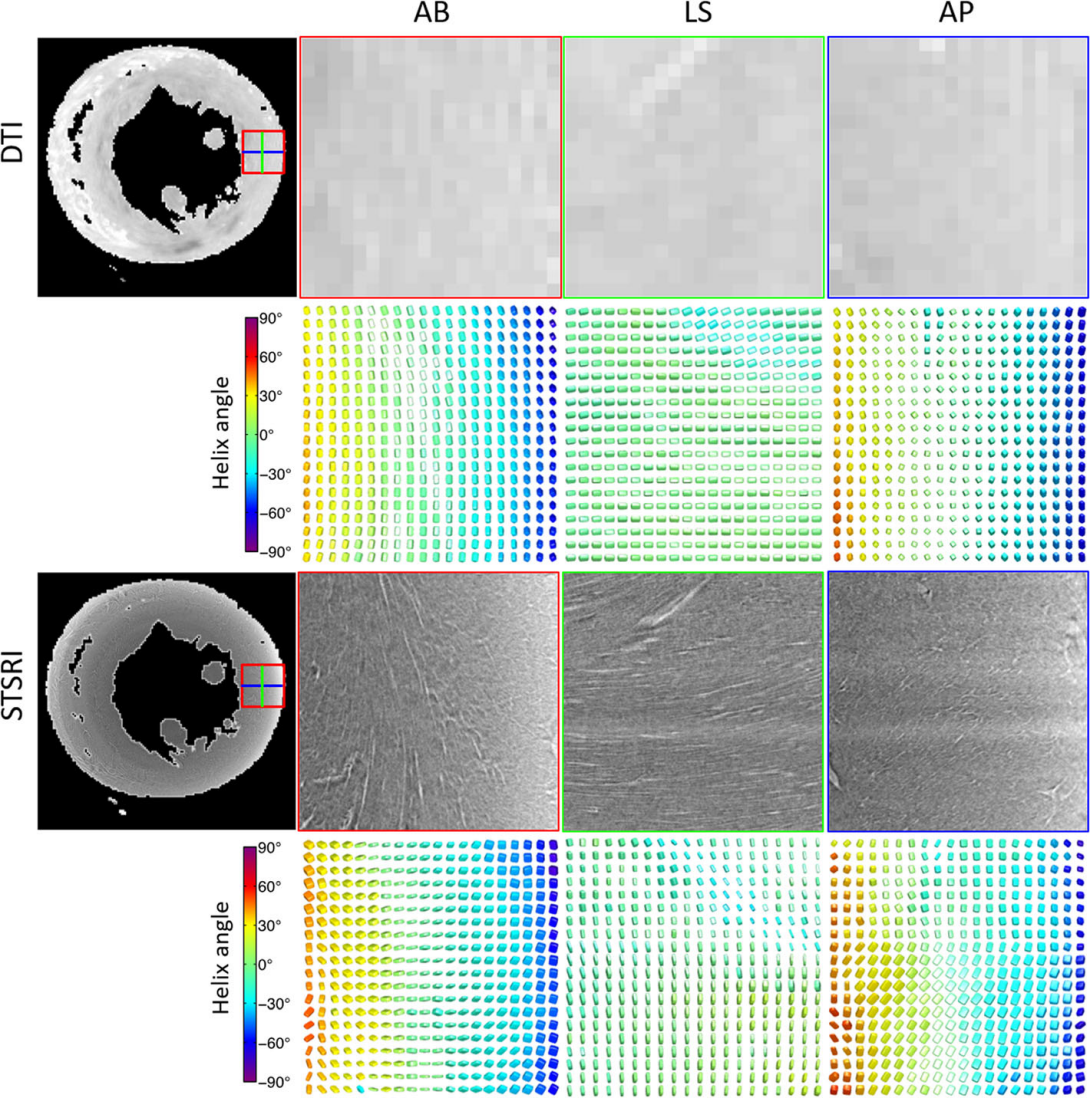
(*Top*) Non-diffusion-weighted image in a mid-ventricular short-axis slice acquired at 100 μm isotropic resolution, including magnified views in the lateral wall of the left ventricle as seen from the apex – base (AB), anterior wall - posterior wall (AP) and lateral wall - septal wall (LS), along with corresponding diffusion tensors. (*Bottom*) Matching SRI images reconstructed at 3.6 μm isotropic resolution reveal cellular (*dark grey*) and extracellular (extracellular fluid and vasculature; *light grey*) structures, along with corresponding structure tensors. Tensors are coloured by helix angle as determined by **v**_**1**,**DT**_ and **v**_**3**,**ST**_

Inspection of the raw images highlights the nominal 28-fold improved resolution in the SRI data relative to the DTI data, and the difference in the contrast mechanisms between the two techniques (Fig. 2). At the SRI reconstructed resolution of 3.6 μm, cells and vessels can be distinguished and are seen to share a common gross alignment. In an example region-of-interest, the tissue volume is comprised primarily of closely packed cardiomyocytes and vessels. Sheetlet structures may be inferred from the regular distribution of small gaps between cell layers. There is a clear transition from left to right helical orientation of cells from the epicardium to endocardium (See Additional file 1). The ST eigenvector **v**_**3,ST**_ is seen to align with the DT eigenvector **v**_**1,DT**_ and the cell long-axis. Residual low-frequency banding artefacts are observed in the SRI data, however these occur on a greater length scale than the ST convolution kernel, and have little impact on estimation of the ST.
**Additional file 1: Video S1.** Transmural synchrotron radiation imaging sequence in the LV lateral wall (lateral-septal view) shows the transition from left to right helical cell long-axis orientations from the subepicardium to the subendocardium (MOV 4080 kb)

### Angle mapping

**Fig. 3:**
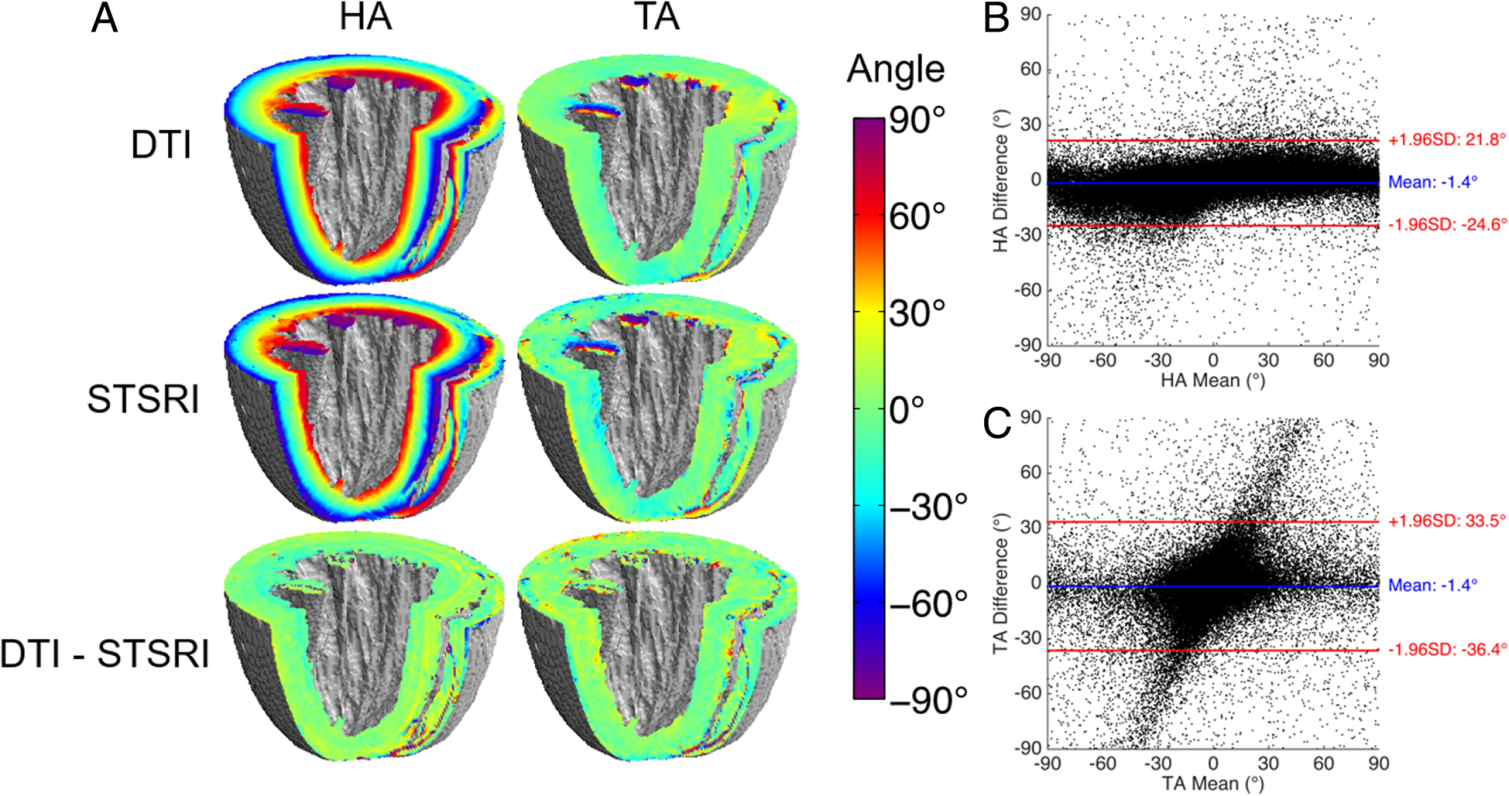
**a** Maps of helix angle (HA) and transverse angle (TA) in whole ex vivo rat heart based on DTI and STSRI, including difference maps; **b**, **c** Bland-Altman plots of HA and TA measured with DTI and STSRI in all voxels in the global myocardium

3D maps of HA and TA in the left and right ventricles show excellent correspondence between the DTI and STSRI data (Fig. 3). In both datasets, a transition in HA from negative to positive values is observed from the epicardium to endocardium, and TA is close to 0° globally. Difference maps indicate good agreement over the majority of the myocardium. Poorer agreement is seen in regions with sudden changes in tensor orientation (eg. mid-ventricular right ventricle wall), and in the neighbourhood of major vessels in the subepicardium. Bland-Altman plots show that HA_DTI-STSRI_ = −1.4° ± 23.2° (mean ± 1.96 standard deviations across all voxels (>6 × 10^5^) in the LV wall) and TA_DTI-STSRI_ = −1.4° ± 35.0°. As TA was close to 0° across the myocardium, any differences between TA_DTI_ and TA_STSRI_ would tend to be double their mean value. This manifested as a y = 2x band of values.

**Fig. 4:**
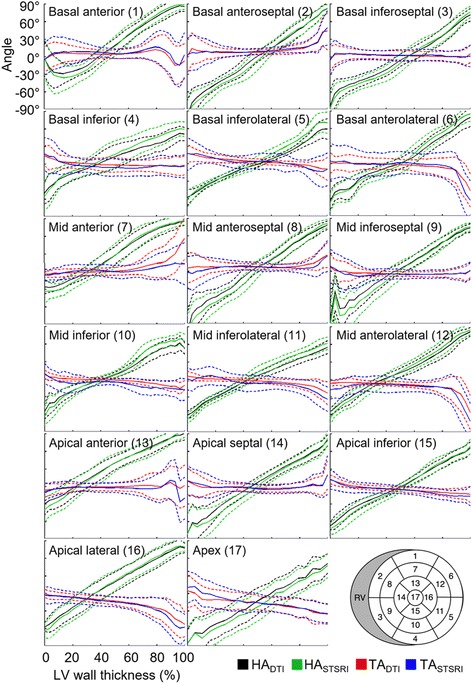
Transmural variation in helix angle (HA) and transverse angle (TA) in global myocardium as measured with DTI and STSRI. Regions were defined by the 17-segment American Heart Association model (*bottom right*). Means (*solid lines*) and standard deviations (*dashed lines*) are given over all voxels in each region. Angles were normalised to wall thickness in each region, with 0% and 100% corresponding to the LV subepicardium and subendocardium respectively

Transmural profiles of HA and TA in the left ventricle based on DTI and STSRI data are presented (Fig. 4) and their linearity and range summarised (Table 1). The profiles show that both the mean and standard deviation of the HA and TA are in good agreement across the different regions and wall depths within the left ventricle. We observed that HA was highly linear across the left ventricle wall, with R^2^ or Linearity_HA,DTI_ ranging from 0.934 to 0.996, Linearity_HA,STSRI_ ranging from 0.920 to 0.997, and Linearity_HA,DTI-STSRI_ = 0.0014 ± 0.0044 (mean ± standard deviation across 17 regions). The distribution of transmural range of HA was proportionally wider than linearity across regions with 93.2° ≤ Range_HA,DTI_ ≤ 166.1°, 106.0° ≤ Range_HA,STSRI_ ≤ 180.1° and Range_HA,DTI-STSRI_ = −12.4° ± 4.4°. The linearity and range of TA was highly variable across regions, but consistent between DTI and STSRI: 0.016 ≤ Linearity_TA,DTI_ ≤ 0.962, 0.001 ≤ Linearity_TA,STSRI_ ≤ 0.937, Linearity_TA,DTI-STSRI_ = 0.096 ± 0.165, −41.1° ≤ Range_TA,DTI_ ≤ 38.2°, −47.9° ≤ Range_TA,STSRI_ ≤ 24.0° and Range_TA,DTI-STSRI_ = 3.9° ± 6.1°.

**Table 1:** Transmural measurements of helix angle (HA) and transverse angle (TA)

ROI	HA (DTI)		HA (STSRI)		TA (DTI)		TA (STSRI)	
	Linearity	Range	Linearity	Range	Linearity	Range	Linearity	Range
1	0.934	131.1	0.920	142.8	0.048	−4.6	0.004	−1.4
2	0.994	166.1	0.996	175.6	0.442	21.2	0.549	24.0
3	0.987	137.7	0.986	148.8	0.227	−3.2	0.140	−2.8
4	0.985	93.2	0.979	106.0	0.693	−12.1	0.403	−12.9
5	0.989	110.2	0.993	126.6	0.962	−21.7	0.890	−26.5
6	0.987	131.9	0.987	148.3	0.509	−24.5	0.388	−31.9
7	0.990	128.4	0.986	139.9	0.658	38.2	0.759	15.7
8	0.988	162.8	0.993	180.1	0.400	10.1	0.382	10.9
9	0.980	146.2	0.979	162.1	0.254	−4.6	0.025	−1.8
10	0.985	110.2	0.981	129.0	0.888	−16.8	0.799	−18.7
11	0.991	111.6	0.987	126.8	0.845	−20.2	0.794	−28.9
12	0.992	127.3	0.997	143.1	0.496	−25.6	0.306	−26.4
13	0.994	124.0	0.990	132.6	0.622	7.0	0.004	−1.4
14	0.995	147.9	0.995	156.5	0.016	1.9	0.001	−0.6
15	0.996	125.9	0.997	137.4	0.924	−20.3	0.931	−23.4
16	0.996	139.5	0.992	149.5	0.843	−41.1	0.811	−47.9
17	0.995	131.9	0.993	131.7	0.927	−31.9	0.937	−40.2

The whole LV was segmented based on the 17-segment American Heart Association model. Linearity and range (°) were measured from the LV subepicardium to subendocardium. Means and standard deviations are reported over all voxels in each region

### Tractography

**Fig. 5:**
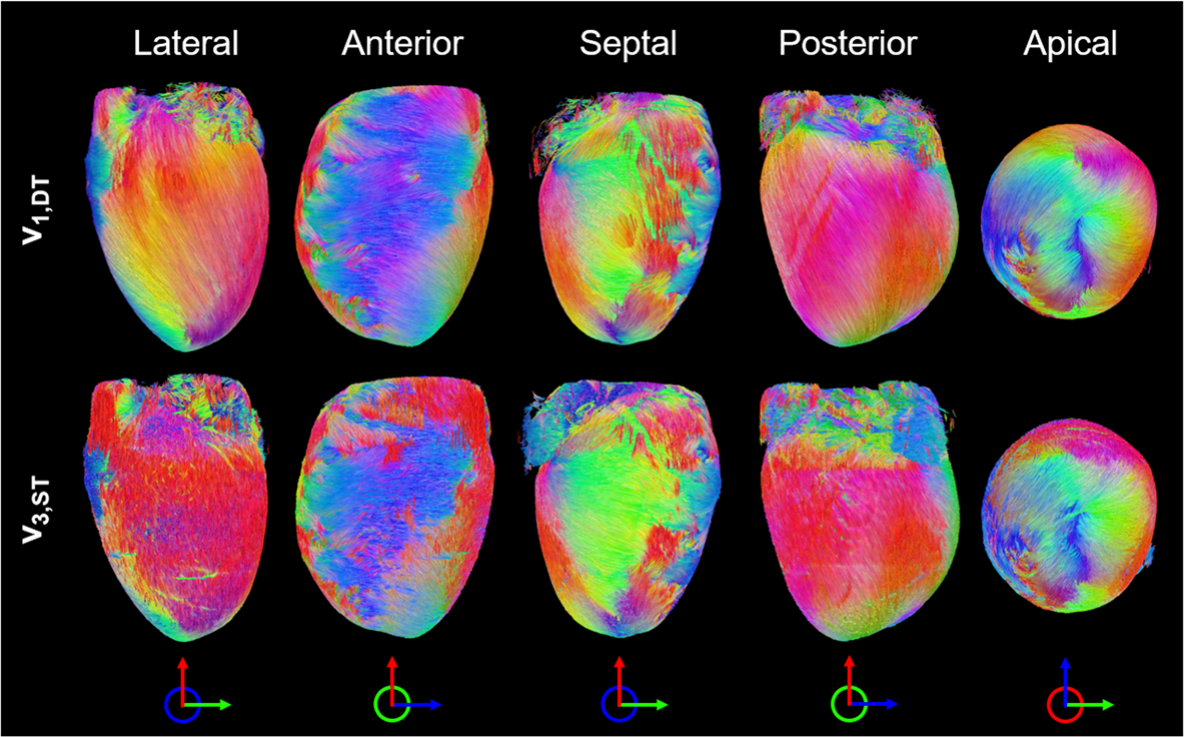
Tracking of eigenvectors corresponding to the cardiomyocyte long-axis in whole heart. **v**_**1**,**DT**_ and **v**_**3**,**ST**_ show excellent correspondence. Tracks are coloured by orientation: apico-basal (*red*), anterior-posterior (*green*) and lateral-septal (*blue*)

**Fig. 6:**
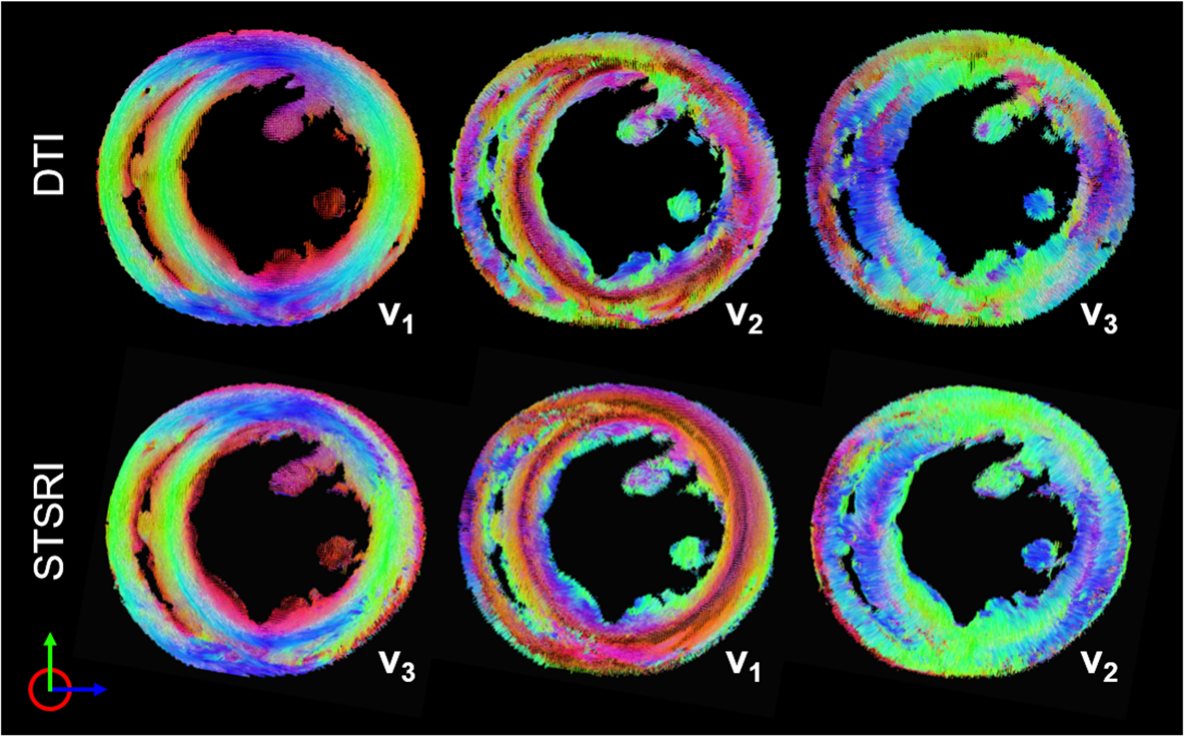
Tracking of principal eigenvectors in a 0.5 mm thick mid-ventricular short-axis slice. Based on STSRI data reconstructed at 3.6 μm isotropic resolution, **v**_**3**,**ST**_, **v**_**1**,**ST**_ and **v**_**2**,**ST**_ generally corresponds with **v**_**1**,**DT**_, **v**_**2**,**DT**_ and **v**_**3**,**DT**_. Tracks are coloured by orientation: apico-basal (*red*), anterior-posterior (*green*) and lateral-septal (*blue*)

Tracks reconstructed based on **v**_**3,ST**_ represent the voxel-averaged cell long-axis orientation (Fig. 5). The left helical orientation of tracks is seen in the lateral and posterior left ventricular subepicardium. Tracks are more circumferential along the anterior left ventricular subepicardium and the right ventricular subepicardium, and spiral in a clockwise manner towards the apex. Slight discontinuities are observed at the subepicardium and at the interfaces between the three concatenated SRI imaging slabs. Tracks reconstructed in a mid-ventricular short-axis slice show agreement of **v**_**1,DT**_, **v**_**2,DT**_ and **v**_**3,DT**_ with **v**_**3,ST**_, **v**_**1,ST**_ and **v**_**2,ST**_ (Fig. 6). This was consistent whether a kernel size of 7^3^ voxels (data not shown) or 11^3^ voxels was used. Discrepancies tend to be localized at the left ventricular subendocardium and the right ventricle. Tracks based on **v**_**3,ST**_ exhibited a transmural variation in HA across the myocardial wall, while tracks based on **v**_**2,ST**_ were generally oriented in a radial manner.

### Sheetlet orientations

To assess confidence in eigenvector sorting, histograms of the ratio of DT and ST eigenvalues were evaluated (See Additional file 2). Putative cell and sheetlet orientations were better discriminated with STSRI, as were putative sheetlet and sheetlet-normal orientations. Mean SE and SA were similar and their mean voxel-wise differences were low, however there was considerable heterogeneity across the myocardium (See Additional file 3).

### Effect of ST reconstruction parameters

**Fig. 7:**
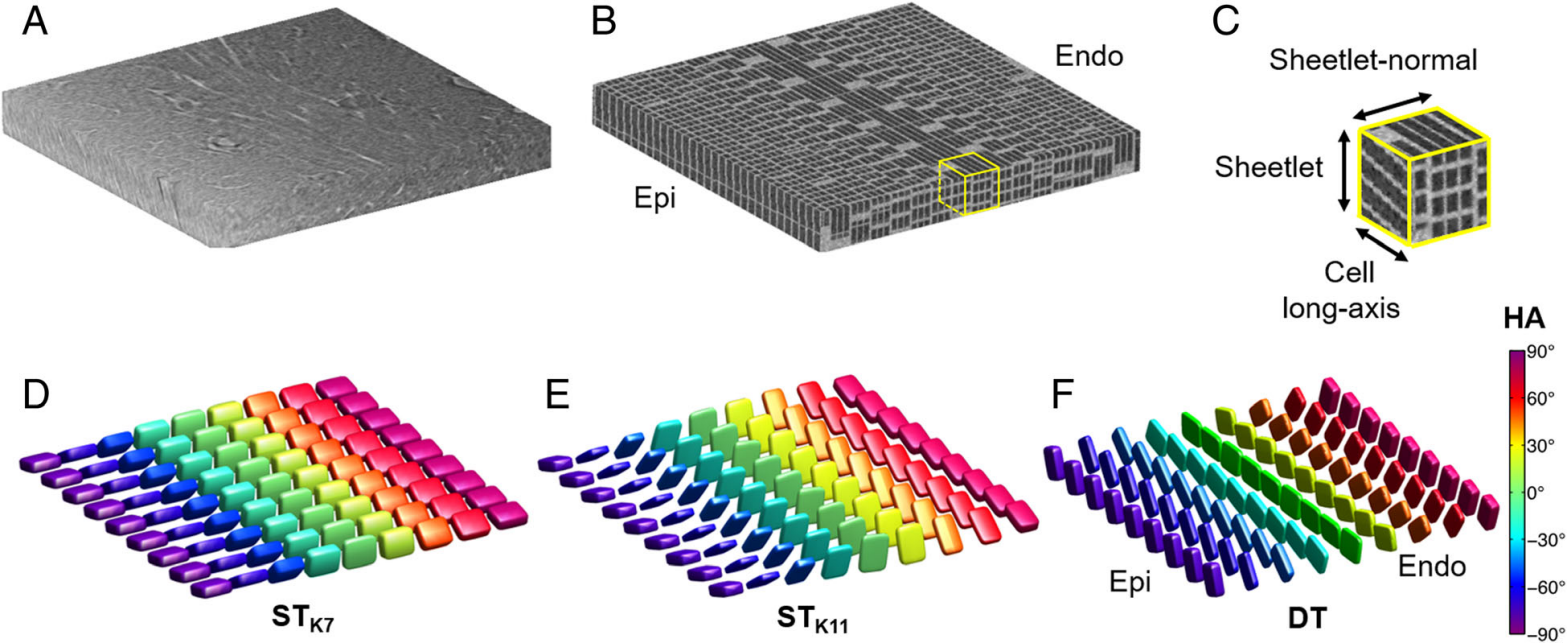
**a** Representative short-axis slice through the LV myocardial wall. **b** Simulated simplified 3D microstructure in a 0.9 × 0.9 × 0.1 mm transmural region in the myocardium. Helix angles (HA) ranged from −88° (subepicardium) to 88° (subendocardium). **c** Magnified 0.1 mm isotropic region showing the simulated cell long-axis, sheetlet and sheetlet-normal directions. **d**, **e** Structure tensors reconstructed at 0.1 mm isotropic resolution with kernel sizes of 7^3^ (K7) and 11^3^ voxels (K11). Tensors are displayed as superquadric glyphs and coloured by helix angle. In both cases, **v**_**3**,**ST**_ is oriented along the voxel-averaged cell long-axis. In contrast, **v**_**1**,**ST**_ is aligned to the sheetlet-normal direction for K7, and the sheetlet direction for K11. **f** Illustration of diffusion tensors

Figure 7 illustrates the correspondence of ST data to a simulated transmural left ventricular volume. Angle estimates closely matched voxel-averaged simulated values: HA_ST_ ranged from −76.9° ± 0.3° (epi) to 76.7° ± 0.5° (endo); TA_ST_ ranged from −0.2° ± 0.7° (epi) to −0.3° ± 1.9° (endo), where the simulated voxel-averaged HA ranged from −77.3° (epi) to 77.3° (endo) and simulated TA = 0°. It was found that the orientation of ST eigenvectors could be modulated by the convolution kernel size. For a kernel size of 7^3^ voxels, **v**_**1,ST**_, **v**_**2,ST**_ and **v**_**3,ST**_ corresponded to the simulated sheetlet-normal, sheetlet and cell long-axis orientations respectively. When the kernel size was 11^3^ voxels, **v**_**1,ST**_, **v**_**2,ST**_ and **v**_**3,ST**_ corresponded to the simulated sheetlet, sheetlet-normal and cell long-axis orientations respectively. The orientation of the STs contrasts with the orientation of DTs where **v**_**1,DT**_, **v**_**2,DT**_ and **v**_**3,DT**_ are understood to generally correspond to the cell long-axis, sheetlet and sheetlet-normal orientations respectively. In practice, this degree of tensor reorientation with ST kernel size is unlikely to occur as biological tissue is more heterogenous and far less coherent.

**Fig. 8:**
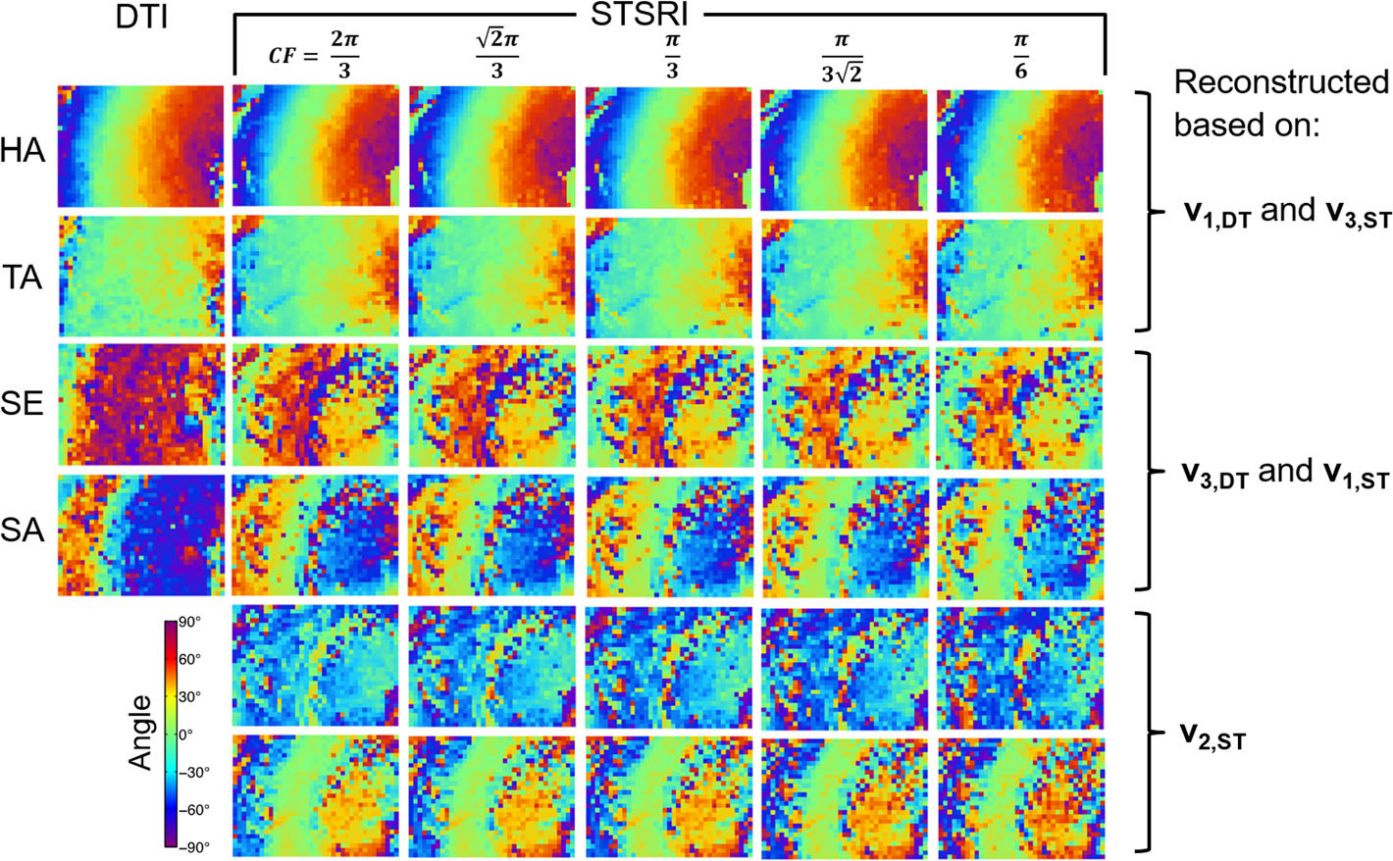
Helix angle (HA), transverse angle (TA), sheetlet elevation (SE) and sheetlet azimuth (SA) in LV myocardium reconstructed with DTI and STSRI using different reconstruction parameters. Here, high-resolution STSRI data with isotropic pixel size of 1.1 μm were analysed using centre frequencies (CF) of $\frac{2\pi}{3},\ \frac{\sqrt{2}\pi}{3},\ \frac{\pi}{3},\ \frac{\pi}{3\sqrt{2}},\ \frac{\pi}{6}$ and corresponding kernel sizes of 7^3^, 7^3^, 9^3^, 11^3^, 17^3^ voxels. The STSRI image corresponding to this region is given in Fig. 9

The impact of ST kernel size and centre frequency on the rat heart data is shown in Fig. 8. STSRI-based estimates of HA and TA are robust to these parameters, while the correspondence of STSRI and DTI-based estimates of SE and SA improved with smaller ST kernel sizes and higher centre frequencies. Contrary to the whole heart STSRI data with a pixel size of 3.6 μm where **v**_**2,ST**_ was found to best correspond with **v**_**3,DT**_, it was observed in this dataset with pixel size of 1.1 μm that **v**_**1,ST**_ best corresponded with **v**_**3**,**DT**_.

### Effect of vasculature

**Fig. 9:**
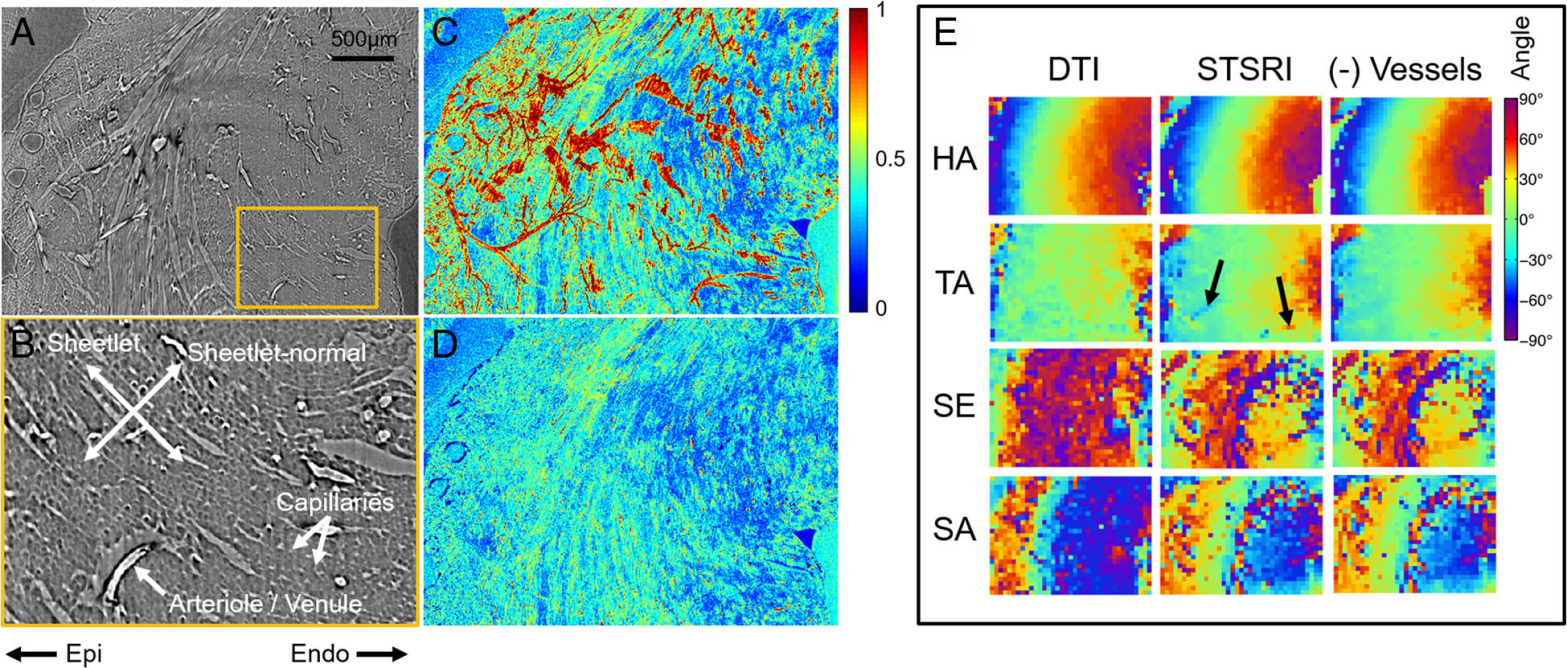
**a** Pilot SRI data in the LV myocardium reconstructed at 1.1 μm isotropic resolution shows improved definition of cells, sheetlets, arterioles, venules and capillaries. Scale bar is 500 μm. **b** Magnified SRI image. **c** Normalised maximum-intensity-projection (MIP) of SRI data through a 100 μm slice, corresponding to a single-voxel thickness in the DTI data, shows bright vascular structures. **d** Normalised MIP of SRI data with vascular component set to 0. **e** Helix angle (HA), transverse angle (TA), sheet elevation (SE) and sheet azimuth (SA) based on DTI, STSRI and STSRI data with vessels excluded. Here, the ST kernel size = 7^3^ voxels, centre frequency = $\frac{2\pi}{3}$, and SE and SA were calculated based on **v**_**3**,**DT**_ and **v**_**1**,**ST**_. Excluding vessels from the ST reconstruction results in modest reduction in heterogeneity due to vessel contribution (see *arrows*)

Pilot SRI data with a pixel size of 1.1 μm shows better cell and sheetlet definition compared to the whole heart data (Fig. 9). Vessels appear bright and can be clearly seen in the maximum intensity projections (MIPs). The vessel segmentation method used was effective as illustrated in the MIP with segmented vessels set to 0. Good correspondence between DTI and STSRI data were observed in angle maps, particularly HA and TA. Modest improvements in smoothness of angle maps were observed when vessels were excluded from the ST reconstruction, particularly where the vessels were most prominent.

## Discussion

The key criteria for validating DTI for assessment of tissue microstructure are that (i) cellular structures can be resolved in three dimensions, (ii) the sample is imaged with both modalities in the same physical state and (iii) validation can be performed in the whole organ. The high brilliance of SRI enables high resolution imaging, while phase retrieval overcomes the intrinsic low soft tissue contrast. Cardiomyocyte and vessel orientation can be distinguished, particularly in the higher resolution SRI data, and their broad mutual alignment is consistent with histological analysis [43]. One striking difference is that the cells and vessels appear much more closely packed compared to typical histological data. The dense packing of structures suggests that the more prominent gaps observed in histological data could be an artefact of cell shrinkage and distortion that is dependent on the sample preparation, with implications on cell size, distribution and volume fraction estimates. Whole heart SRI data was acquired in 10 h. This is considerably faster and less user-dependent than histological preparation and imaging of much smaller regions.

### Cardiomyocyte orientations

Our results show that **v**_**3**,**ST**_ corresponds to **v**_**1**,**DT**_ and the dominant cell long-axis. HA and TA estimates from the DTI and STSRI data were in remarkable agreement across the myocardium. The known left to right helical arrangement of cells from the left ventricular subepicardium to subendocardium is observed in both DTI and STSRI data in all 17 regions, with similar transmural linearities and ranges. We find that **v**_**3**,**ST**_ tracks tend to be shorter than **v**_**1**,**DT**_ tracks, and there appear to be missing **v**_**3**,**ST**_ tracks in the mid-ventricular right ventricular subepicardium. This can be attributed to the amplification of phase differences at tissue interfaces at major vessels and the surface of the heart, leading to enhanced contribution of such structures to the ST. Boundary effects at the heart surface lead to overestimation of **v**_**1**,**ST**_ perpendicular to the wall surface that can overshadow the contribution of sheetlet orientations. These effects can be mitigated by smoothing of signal intensities at the heart surface. These findings reinforce the observation that **v**_**1**,**DT**_ corresponds to the dominant cardiomyocyte long-axis [14, 20–22, 44], and extends this observation across the whole heart.

### Sheetlet orientations

Resolution of sheetlet and sheetlet-normal orientations is complicated in SRI by the dense packing of cells, which can make it difficult to positively identify sheetlets. In the whole heart data, we observed general correspondence of **v**_**1**,**ST**_ and **v**_**2**,**ST**_ to **v**_**2**,**DT**_ and **v**_**3**,**DT**_ respectively. This would appear counterintuitive as **v**_**1**,**ST**_ measures the normal to planar structures, and therefore may be expected to correspond to **v**_**3**,**DT**_. This suggests that the estimation of **v**_**1**,**ST**_ and **v**_**2**,**ST**_ may be influenced by factors other than sheetlet organisation. One likely contributor is the highly organised vascular network. Moreover, the distribution of sheetlets is heterogeneous across the myocardium. This manifests in changing length scales over which sheetlets occur, and could affect the interpretation of sheetlets in STSRI. In contrast, we observed in the higher-resolution SRI data that **v**_**2**,**ST**_ and **v**_**1**,**ST**_ generally corresponded to **v**_**2**,**DT**_ and **v**_**3**,**DT**_ respectively. The higher-resolution SRI images are characterised by better defined cells and sheetlets that reduce the influence of the microvasculature relative to the whole heart SRI images. Exclusion of the vasculature from the ST reconstruction in the higher-resolution SRI data confirmed that the vascular contribution to the estimated angle maps was relatively minimal. This suggests that the estimation of cell and sheetlet orientations may be primarily governed by the spatial resolution and relative dominance of structural features in the SRI images. Few histological validation studies have investigated sheetlets, and these are affected by cell shrinkage and tissue distortion. In one study, sheetlet populations were identified on the basis of large gaps between groups of cells [44]. These gaps appear to be the result of preparation and they manifest non-uniformly in the myocardium. Further limitations were small fields-of-view, difficulty in registration and subjectivity in assessing sheetlet structures. Another study imaged ink prints of unfixed heart thus avoiding preparation artefacts [20]. However, the study faced issues of imprecise registration, limited field-of-view (FOV), and extrapolation of 3D sheetlet orientations from 2D data. Validation of DTI with anatomical MRI and ST analysis showed good correspondence of **v**_**1**,**DT**_, **v**_**2**,**DT**_ and **v**_**3**,**DT**_ with **v**_**3**,**ST**_, **v**_**2**,**ST**_ and **v**_**1**,**ST**_ [27]. This is consistent with our higher-resolution data. However, the resolution of the anatomical MRI data was insufficient to resolve cellular structures. Image contrast would have depended additionally on the net signal contribution of sub-voxel structures including cells, extracellular fluid and vessels with different T_2_*. Regional variation in correspondence of putative sheetlet and sheetlet-normal directions between DT and ST was observed, consistent with our findings. Further work is needed to confirm correspondence of DT eigenvectors with sheetlet structures. Equally important to consider are the many factors in DTI, as described in *Background*, that could lead to errors in eigenvector sorting and assessment of microstructure if not properly addressed.

### Study considerations

There are several technical aspects to consider in STSRI. First, tissue fixation with high osmolality fixatives can lead to cell shrinkage. This was mitigated by the use of isosmotic fixative. The acquisition time could be shortened by reducing exposure times, while the image quality of the SRI data could be further improved by increasing the imaging resolution at the cost of FOV and scan time. This helps disambiguate cells, sheetlets and capillaries with greater confidence. As with other imaging methods, there are invariably tradeoffs between spatial resolution and coverage in SRI, and imaging whole hearts in larger species would be difficult. Imaging of intact whole human hearts at cellular resolution is not currently feasible, and this drives the need for validation studies in small animal models. Furthermore, validation using ex vivo samples enables acquisition of high quality structural information, free of confounds such as motion, strain and perfusion. Although the sample size was small, this is comparable to histological validation studies of DTI [21, 22]. Moreover, our study assessed correspondence of DTI and STSRI measurements across over 600,000 myocardial voxels, far exceeding existing studies.

Phase-contrast imaging exploits the refraction of X-rays by a sample instead of its absorption as in conventional X-ray imaging, yielding superior image contrast in samples with small differences in density. The phase retrieval method we used relies on the assumptions that the sample comprises a single homogeneous material with fixed *δ*_*λ*_/*β*_*λ*_, monochromatic X-rays are used and the propagation distance is in the near-field. Here, a polychromatic beam was used in heterogeneous tissue in the near field. The use of a polychromatic beam and single *δ*_*λ*_/*β*_*λ*_ value results in non-quantitative values of the reconstructed phase shift. However, there was ample contrast for identification of structures via ST analysis. As materials have different *δ*_*λ*_/*β*_*λ*_ values, imaging of heterogeneous tissue leads to unavoidable edge enhancement fringes and blurring, particularly at the interface of tissues with marked differences in *δ*_*λ*_/*β*_*λ*_. We identified *δ*_*λ*_/*β*_*λ*_ following optimisation based on maximising image contrast and minimising these artefacts. In future work, we plan to explore the use of a multi-layer monochromator, which has the added benefit of reducing sample exposure to X-rays. Residual artefacts, such as banding or ringing, may arise due to imperfections or non-linear responses in the detector or scintillation screen. These can be mitigated with improvements in hardware and filtering.

Another consideration is the convolution kernel used in the ST analysis, which affects the size of structures to which the reconstruction is sensitive. A fixed kernel presupposes that structures are regularly sized and that structures are sufficiently anisotropic for reliable eigenvalue separation. In the highly simplified simulation of coherently organised cuboidal cells, a variation in kernel size was shown to be sufficient to sensitise the ST reconstruction to different microstructural features, leading to swapping of **v**_**2**,**ST**_ and **v**_**3**,**ST**_. This effect would be much less prominent in biological tissues with greater structural heterogeneity. Indeed, varying the kernel size from 7^3^ to 11^3^ voxels did not affect the orientations of **v**_**2**,**ST**_ and **v**_**3**,**ST**_ in the whole heart data. While the kernel size was seen to influence the estimation of angle maps in the higher-resolution SRI data, the effect was relatively modest. It is therefore important that the kernel be optimised based on the specific microstructure, particularly in the case of pathological tissue. It may also be possible to extract information from structures with different characteristic length scales by varying the kernel. Finally, the current reconstructed ST resolution was chosen to match the DTI resolution, with each output ST calculated based on averaging of 28^3^ = 21952 voxels from the SRI images. It may be feasible to obtain robust measurements with far less averaging, thereby increasing the final isotropic resolution of the ST data to < 50 μm. This translates to voxels containing ~10 cells, greatly diminishing the contribution of cell dispersion and heterogeneity to the ST.

## Conclusions

DTI is a unique non-invasive method that enables inference of 3D tissue structure in-vivo. It is however sensitive to a wide range of physical and biological processes, and validation is vital to improving our understanding of DTI, and its acceptance in clinical practice. Here, we performed the first validation of DTI across the whole intact heart, and without additional preparation following DTI. STSRI affirmed the correspondence of **v**_**1**,**DT**_ with the dominant cardiomyocyte long-axis in the whole heart, and suggests that **v**_**2**,**DT**_ and **v**_**3**,**DT**_ generally correspond to the sheetlet and sheetlet-normal orientations, corroborating histological findings and independently informing the interpretation of cardiac DTI. STSRI opens up exciting possibilities in 3D quantification of cell morphology in large fields-of-view without distortion, augmentation of structure-based electro-mechanical modeling, validation of in vivo contrast-enhanced imaging measurements of extracellular volume, and validation of advanced diffusion MRI methods for assessing biophysical parameters such as cell size, shape and dispersion. The next step would be to use STSRI to improve characterisation of abnormal myocardium, and to validate DTI measurements in cardiac disease such as myocardial infarction and hypertrophy.

## Additional files

Additional file 2: Figure S1. Histograms of ratios of principal eigenvalues (PDF 238 kb)
Additional file 3: Figure S2. Sheetlet elevation and azimuth maps and histograms (PDF 610 kb)

## Abbreviations

2D: Two-dimensional
3D: Three-dimensional
DT: Diffusion tensor
DTI: Diffusion tensor imaging
DW: Diffusion-weighted
HA: Helix angle
LV: Left ventricle
MIP: Maximum intensity projection
MRI: Magnetic resonance imaging
RV: Right ventricle
SRI: Synchrotron radiation imaging
ST: Structure tensor
STSRI: Structure tensor synchrotron radiation imaging
TA: Transverse angle
