# Supplementary material for: Validation of diffusion tensor MRI measurements of cardiac microstructure with structure tensor synchrotron radiation imaging
Source: J Cardiovasc Magn Reson. 2017 Mar 10;19:31. doi: 10.1186/s12968-017-0342-x (PMC5345150; doi:10.1186/s12968-017-0342-x)
Supplement: Additional file 2: Figure S1. — Histograms of ratios of principal eigenvalues (PDF 238 kb) [file 12968_2017_342_MOESM2_ESM.pdf]

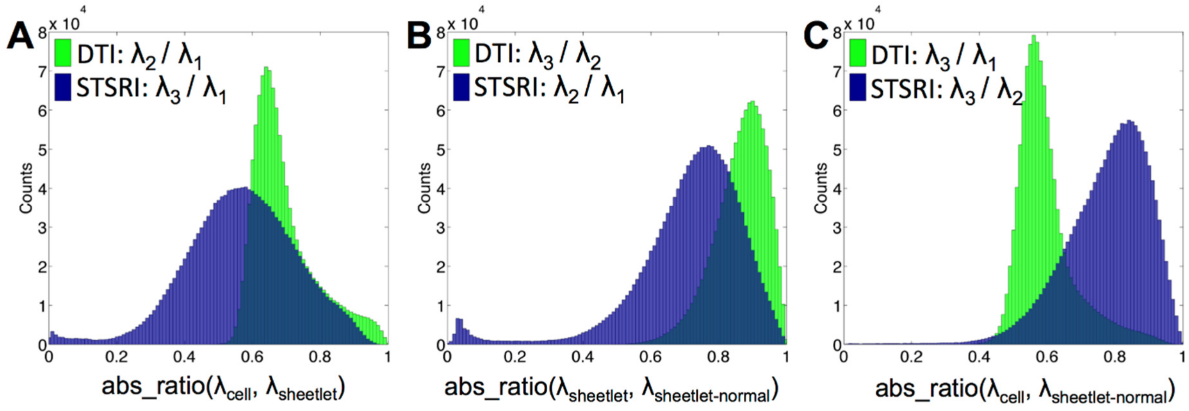

Figure S1. Histograms of ratios of principal eigenvalues. STSRI data were reconstructed at 3.6 μm isotropic resolution. (A) abs_ratio(λ_{cell}, $\lambda_{sheetlet}$), (B) abs_ratio($\lambda_{sheetlet}$, $\lambda_{sheetlet-normal}$) and (C) abs_ratio($\lambda_{cell}$, $\lambda_{sheetlet-normal}$) in diffusion tensor imaging (DTI) and structure tensor synchrotron radiation imaging (STSRI), where abs_ratio(λ_i, λ_j) = { λ_i/λ_j if $\lambda_i \leq \lambda_j$ or λ_j/λ_i if $\lambda_i > \lambda_j$. Eigenvalues are better separated in the STSRI data corresponding to the (i) putative cell and sheetlet orientations: $(\lambda_2/\lambda_1)_{DT, mean} = 0.69$ versus $(\lambda_3/\lambda_1)_{ST, mean} = 0.58$, and (ii) putative sheetlet and sheetlet-normal orientations: $(\lambda_3/\lambda_2)_{DT, mean} = 0.86$ versus $(\lambda_2/\lambda_1)_{ST, mean} = 0.71$. Eigenvalues are better separated in the DTI data corresponding to putative cell and sheetlet-normal orientations: $(\lambda_3/\lambda_1)_{DT, mean} = 0.60$ versus $(\lambda_3/\lambda_2)_{ST, mean} = 0.78$.
