# Supplementary material for: Validation of diffusion tensor MRI measurements of cardiac microstructure with structure tensor synchrotron radiation imaging
Source: J Cardiovasc Magn Reson. 2017 Mar 10;19:31. doi: 10.1186/s12968-017-0342-x (PMC5345150; doi:10.1186/s12968-017-0342-x)
Supplement: Additional file 3: Figure S2. — Sheetlet elevation and azimuth maps and histograms (PDF 610 kb) [file 12968_2017_342_MOESM3_ESM.pdf]

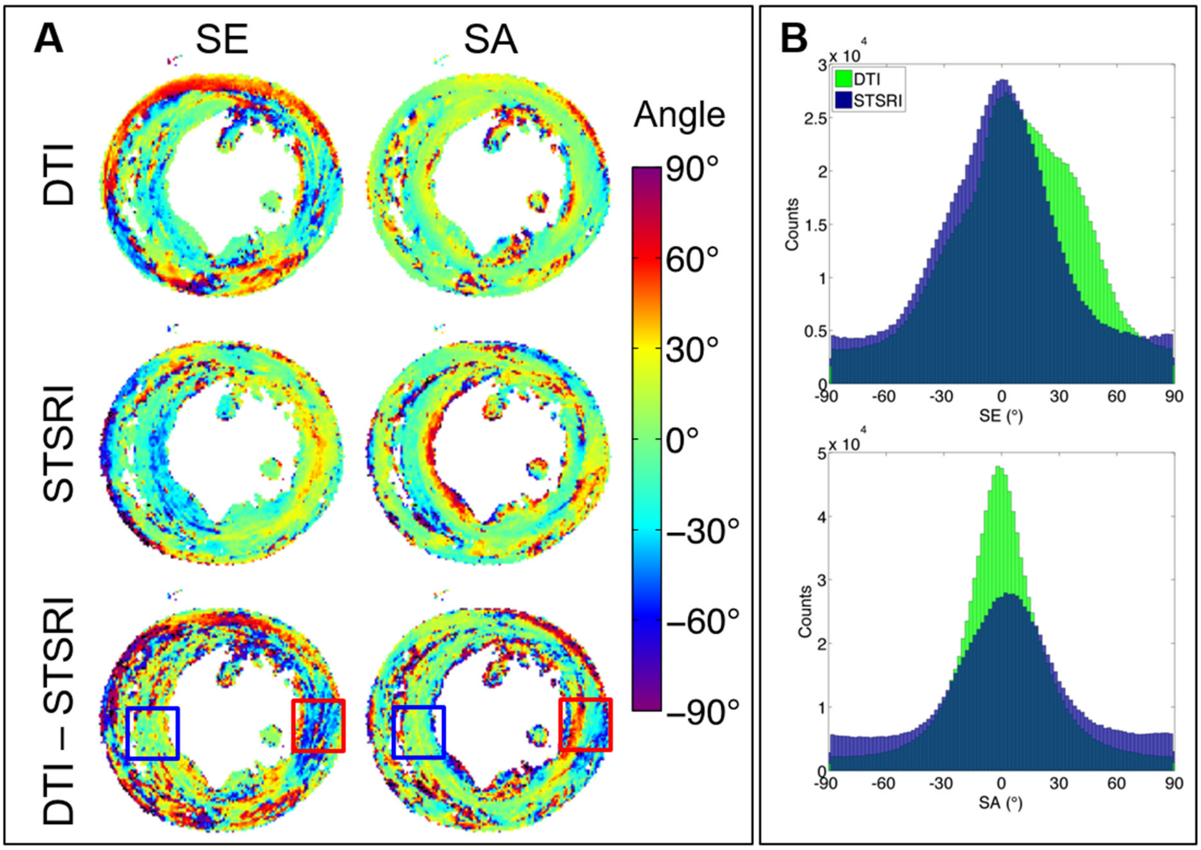

Figure S2. Sheetlet elevation and azimuth maps and histograms. (A) Sheetlet elevation (SE) and sheetlet azimuth (SA) maps based on $\mathbf{v}_{3,DT}$ and $\mathbf{v}_{2,ST}$ in a mid-ventricular short-axis slice. STSRI data were reconstructed at 3.6 µm isotropic resolution. SE was more longitudinally oriented ($|SE| > 60°$) in the anterior and posterior left ventricular subepicardium and right ventricular subepicardium in the DTI data, and the right ventricular subepicardium in the STSRI data. SA was generally radial ($|SA| < 30°$) in both DTI and STSRI data. However, it was more circumferential in the anterolateral left ventricular subepicardium in the DTI data, and the septal left ventricular subendocardium in the STSRI data. Difference maps show regions of relative good (blue boxes) and poor (red boxes) correspondence between the two imaging modalities. (B) Histograms of SE and SA based on DTI and STSRI measurements show that SE was higher in DTI compared to STSRI: $SE_{DT} = 6.7° \pm 35.7°$, $SE_{ST} = -0.8° \pm 36.5°$ (mean ± standard deviation over all voxels in the myocardium). Mean SA was similar: $SA_{DT} = 0.5° \pm 28.6°$, $SA_{ST} = 2.2° \pm 38.6°$, but there were heavier tails in the STSRI data. While mean voxel-wise differences between DTI and STSRI were low, there was a considerable spread of angular differences: $SE_{DTI\text{-}STSRI} = 4.7° \pm 46.3°$ and $SA_{DTI\text{-}STSRI} = -2.3° \pm 43.8°$.
